# Synthesis, biological evaluation, and molecular docking of novel hydroxyzine derivatives as potential AR antagonists

**DOI:** 10.3389/fchem.2022.1053675

**Published:** 2022-11-03

**Authors:** Yueheng Qi, Baoli Xue, Shijin Chen, Wang Wang, Haifeng Zhou, Hong Chen

**Affiliations:** ^1^ Luoyang Key Laboratory of Organic Functional Molecules, College of Food and Drug, Luoyang Normal University, Luoyang, Henan, China; ^2^ Hubei Key Laboratory of Natural Products Research and Development, College of Biological and Pharmaceutical Sciences, China Three Gorges University, Yichang, China

**Keywords:** hydroxyzine derivatives, cytotoxic activity, antagonistic activity, docking study, AR antagonists

## Abstract

Prostate cancer (PCa) is a malignant tumor with a higher mortality rate in the male reproductive system. In this study, the hydroxyazine derivatives were synthesized with different structure from traditional anti-prostate cancer drugs. In the evaluation of *in vitro* cytotoxicity and antagonistic activity of PC-3, LNCaP, DU145 and androgen receptor, it was found that the mono-substituted derivatives on the phenyl group (**4**, **6**, **7**, and **9**) displayed strong cytotoxic activities, and compounds **11**–**16** showed relatively strong antagonistic potency against AR (Inhibition% >55). Docking analysis showed that compounds **11** and **12** mainly bind to AR receptor through hydrogen bonds and hydrophobic bonds, and the structure-activity relationship was discussed based on activity data. These results suggested that these compounds may have instructive implications for drug structural modification in prostate cancer.

## 1 Introduction

Targeted anti-tumor drugs are the focus of modern anti-cancer research, because of their special targeting, they can greatly reduce the toxicity to normal cells (Falciani et al., 2010). In addition, cells in rapid division were more sensitive to the most drugs due to the differences in cell dynamics (Tannock, 1978). Therefore, targeted antitumor drugs can simultaneously inhibit the proliferation and differentiation of tumor cells and greatly accelerate the death of tumor cells (Vinaya et al., 2011). Prostate cancer (PCa) is a malignant tumor with a higher mortality rate in the male reproductive system (Greenlee et al., 2001). Prostate cancer is driven by the androgen receptor (Yap et al., 2016; Dai et al., 2017), and is directly associated with nuclear steroidal AR (Bentel and Tilley, 1996; Culig et al., 2002; Gelmann, 2002). Androgens bind to the AR and form a hormone-receptor complex, which can bind to the DNA and induce downstream biological effects (Dehm and Tindall, 2007). This complex also induces the proliferation of prostate cells, and ultimately causes tumorigenesis (Heinlein and Chang, 2004). Although some current treatments (hormonotherapy, radical prostatectomy, chemotherapy, or local radiotherapy) can treat androgen-dependent prostate cancer (Frydenberg et al., 1997). However, drug resistance problems hinder its therapeutic efficacy. Therefore, early detection and elimination of both types of prostate cancer cells are very important for decreasing prostate cancer-related death (Feldman and Feldman, 2001).

**FIGURE 1:**
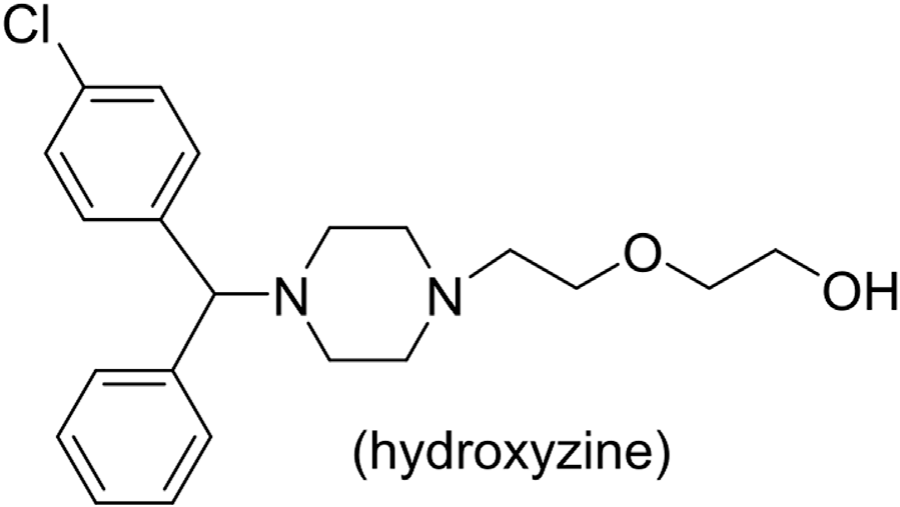
Structures of hydroxyzine.

**SCHEME 1:**
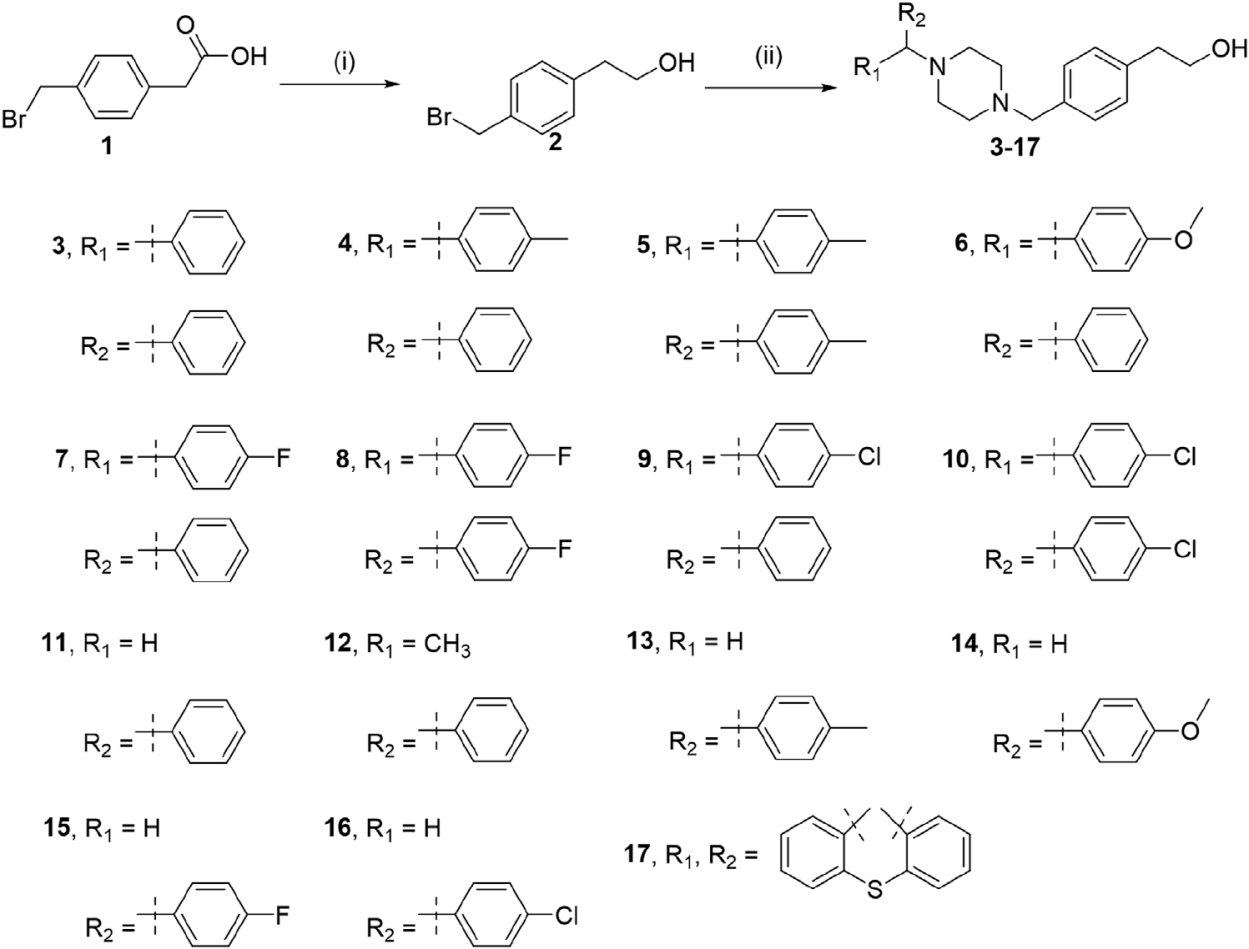
Reagents and conditions: (i) BH_3_.S(CH_3_)_2_, THF, 0°C for 1 h, and room temperature for 10 h; (ii) Piperazines, K_2_CO_3_, CH_3_CN, reflux, 16 h.

Piperazine moieties play an important role in many drugs (Chaudhary et al., 2006), and piperazine derivatives also have exhibited biological importance, such as receptor high-affinity properties (Leopoldo et al., 2007; Romeiro et al., 2011; Chen et al., 2012; Ananthan et al., 2014; Baran et al., 2014) and anti-proliferative properties (Berardi et al., 2008; Lee et al., 2010; Abate et al., 2011; Cao et al., 2013; Liu et al., 2013; Arnatt et al., 2014; Guo et al., 2015). Hydroxyzine (Figure 1) has antihistamine effect and can be quickly absorbed and distributed by oral or muscle injection. The arylpiperazine derivatives were reported to exhibit significant antagonism against AR with an IC_50_ of 0.11 μM, whereas the IC_50_ of bicaluramide is 50 μM. Results of animal experiments have shown that the mass of prostate in rats is significantly reduced, and the concentration of serum testosterone is not significantly changed (Kinoyama et al., 2004; Kinoyama et al., 2005; Gupta et al., 2016). However, there have been few studies on hydroxyzine derivatives. Based on the results of our group’s previous anti-prostate cancer study (Chen et al., 2016; Chen et al., 2017; Chen et al., 2018a; Chen et al., 2019a; Chen et al., 2019b; Qi et al., 2022), we tried to design and synthesize a series of novel hydroxyzine derivatives (Scheme 1) with 2-p-tolylethanol group instead of 2-ethoxyethanol group in hydroxyzine. Unexpectedly, some derivatives exhibited strong anti-cancer activities and antagonistic activities. These results can provide valuable information for further designing hydroxyzine derivatives as potential prostate cancer therapeutics.

## 2 Materials and methods

### 2.1 Materials and instruments

Reagents and solvents were of analytical purity and dried and using standard procedures. The melting point was measured using the Shanghai electrical optical SGW X-4 micromelting point instrument. The HRMS mass spectrometry was measured using the LCQ DECA XP LC-MS. The NMR spectra were measured using the Bruker AV-400 NB, with TMS as the internal standard, and DMSO-*d*_6_ or CDCl_3_ as the solvent. Column chromatography silica gel was the 300–400 mesh silicone of Qingdao Marine Chemical Plant.

### 2.2 Synthesis of 2-[4-(bromomethyl)phenyl]ethanol (2)

The borane–dimethyl sulfide (20.0 ml, 0.038 mol, 2 M in THF) was added to the tetrahydrofuran (THF, 100 ml) solution, supplemented with carboxylic acid **1** (5 g, 0.021 mol), and stirred at 0°C for 1 h. Then stirred at r. t. for 10 h. Extracted with ethyl acetate (100 ml) and water (20 ml). Concentrated organic phase, the resulting residue was directly used without further purification.

### 2.3 Preparation of derivatives 3–17

Piperazines (1.3 equiv), potassium carbonate (5.5 equiv), acetonitrile (CH_3_CN, 10 ml), and **2** (50 mg, 0.11 mmol) were successively added to the flask, stirred with reflux for 10 h. The reaction solution was filtered and concentrated, and purified by silica gel column chromatography (ethyl acetate/petroleum ether = 1/5).

#### 2.3.1 2-(4-((4-benzhydrylpiperazin-1-yl)methyl)phenyl)ethan-1-ol (**3**)

White solid (ethyl acetate), yield: 85% (from compound **1**); M.p. 122°C–123°C; ^1^H NMR (400 MHz, DMSO-*d*_6_) δ in ppm: 7.75–7.10 (m, 14H), 4.26 (br s, 2H), 3.64 (br s, 10H), 3.56 (t, *J* = 6.9 Hz, 2H), 2.70 (t, *J* = 6.9 Hz, 2H); MS (ESI, m/z): 387.1 [M+1]^+^; HRMS (EI) calcd for C_26_H_29_ClN_2_O, 386.2358; found, 386.2354.

#### 2.3.2 2-(4-((4-(phenyl(p-tolyl)methyl)piperazin-1-yl)methyl)phenyl)ethan-1-ol (**4**)

White solid (ethyl acetate), yield: 82% (from compound **1**); M.p. 125°C–126°C; ^1^H NMR (400 MHz, DMSO-*d*_6_) δ in ppm: 7.80–7.11 (m, 13H), 4.27 (br s, 2H), 3.66 (br s, 10H), 3.57 (t, *J* = 6.9 Hz, 2H), 2.72 (t, *J* = 6.9 Hz, 2H); 2.34 (s, 3H), MS (ESI, m/z): 401.2 [M+1]^+^; HRMS (EI) calcd for C_27_H_32_N_2_O, 400.2515; found, 400.2510.

#### 2.3.3 2-(4-((4-(di-p-tolylmethyl)piperazin-1-yl)methyl)phenyl)ethan-1-ol (**5**)

White solid (ethyl acetate), yield: 82% (from compound **1**); M.p. 128°C–129°C; ^1^H NMR (400 MHz, DMSO-*d*_6_) δ in ppm: 7.75 (br s, 4H), 7.45 (d, *J* = 8.0 Hz, 2H), 7.23 (d, *J* = 8.0 Hz, 2H), 7.16 (t, *J* = 8.1 Hz, 4H), 4.25 (br s, 2H), 3.64 (br s, 10H), 3.56 (t, *J* = 6.9 Hz, 2H), 2.71 (t, *J* = 6.9 Hz, 2H); 2.36 (s, 6H), MS (ESI, m/z): 415.1 [M+1]^+^; HRMS (EI) calcd for C_28_H_34_N_2_O, 414.2671; found, 414.2668.

#### 2.3.4 2-(4-((4-((4-methoxyphenyl)(phenyl)methyl)piperazin-1-yl)methyl)phenyl)ethan-1-ol (**6**)

White solid (ethyl acetate), yield: 87% (from compound **1**); M.p. 116°C–117°C; ^1^H NMR (400 MHz, DMSO-*d*_6_) δ in ppm: 7.76–7.13 (m, 13H), 4.25 (br s, 2H), 3.77 (s, 3H), 3.67 (br s, 10H), 3.54 (t, *J* = 6.9 Hz, 2H), 2.74 (t, *J* = 6.9 Hz, 2H), MS (ESI, m/z): 417.1 [M+1]^+^; HRMS (EI) calcd for C_27_H_32_N_2_O_2_, 416.2464; found, 416.2462.

#### 2.3.5 2-(4-((4-((4-fluorophenyl)(phenyl)methyl)piperazin-1-yl)methyl)phenyl)ethan-1-ol (**7**)

White solid (ethyl acetate), yield: 87% (from compound **1**); M.p. 116°C–117°C; ^1^H NMR (400 MHz, DMSO-*d*_6_) δ in ppm: 7.70–7.11 (m, 13H), 4.27 (br s, 2H), 3.64 (br s, 10H), 3.57 (t, *J* = 6.9 Hz, 2H), 2.72 (t, *J* = 6.9 Hz, 2H), MS (ESI, m/z): 405.1 [M+1]^+^; HRMS (EI) calcd for C_26_H_29_FN_2_O, 404.2264; found, 404.2260.

#### 2.3.6 2-(4-((4-(bis(4-fluorophenyl)methyl)piperazin-1-yl)methyl)phenyl)ethanol (**8**)

White solid (ethyl acetate), yield: 70% (from compound **1**); M.p. 133°C–134°C; ^1^H NMR (400 MHz, DMSO-*d*_6_) δ in ppm: 1H NMR (400 MHz, DMSO-d6) δ 7.70 (br s, 4H), 7.46 (d, *J* = 8.0 Hz, 2H), 7.22 (d, *J* = 8.0 Hz, 2H), 7.18 (t, *J* = 8.2 Hz, 4H), 4.27 (br s, 2H), 3.66 (br s, 10H), 3.55 (t, *J* = 6.9 Hz, 2H), 2.68 (t, *J* = 6.9 Hz, 2H); MS (ESI, m/z): 423.2 [M+1]^+^; HRMS (EI) calcd for C_26_H_28_F_2_N_2_O, 422.2170; found, 422.2168.

#### 2.3.7 2-(4-((4-((4-chlorophenyl)(phenyl)methyl)piperazin-1-yl)methyl)phenyl)ethanol (**9**)

White solid (ethyl acetate), yield: 75% (from compound **1**); M.p. 130°C–131°C; ^1^H NMR (400 MHz, DMSO-*d*_6_) δ in ppm: 7.89–7.13 (m, 13H), 4.29 (br s, 2H), 3.66 (br s, 10H), 3.57 (t, *J* = 6.9 Hz, 2H), 2.71 (t, *J* = 6.9 Hz, 2H); MS (ESI, m/z): 421.1 [M+1]^+^; HRMS (EI) calcd for C_26_H_29_ClN_2_O, 420.1968; found, 420.1962.

#### 2.3.8 2-(4-((4-(bis(4-chlorophenyl)methyl)piperazin-1-yl)methyl)phenyl)ethan-1-ol (**10**)

White solid (ethyl acetate), yield: 72% (from compound **1**); M.p. 127°C–128°C; ^1^H NMR (400 MHz, DMSO-*d*_6_) δ in ppm: 7.67 (br s, 4H), 7.42 (d, *J* = 8.0 Hz, 2H), 7.23 (d, *J* = 8.0 Hz, 2H), 7.16 (t, *J* = 8.0 Hz, 4H), 4.23 (br s, 2H), 3.68 (br s, 10H), 3.57 (t, *J* = 6.9 Hz, 2H), 2.66 (t, *J* = 6.9 Hz, 2H); MS (ESI, m/z): 455.2 [M+1]^+^; HRMS (EI) calcd for C_26_H_28_Cl_2_N_2_O, 454.1579; found, 454.1575.

#### 2.3.9 2-(4-((4-benzylpiperazin-1-yl)methyl)phenyl)ethan-1-ol (**11**)

White solid (ethyl acetate), yield: 82% (from compound **1**); M.p. 119°C–120°C; ^1^H NMR (400 MHz, CDCl_3_) δ in ppm: 7.43 (d, *J* = 8.0 Hz, 2H), 7.32 (t, *J* = 8.0 Hz, 2H), 7.24 (d, *J* = 8.0 Hz, 2H), 7.05 (d, *J* = 7.8 Hz, 2H), 6.98 (t, *J* = 7.6 Hz, 1H), 4.25 (s, 2H), 3.76 (t, *J* = 6.8 Hz, 2H), 3.62 (s, 2H), 3.54 (t, *J* = 5.0 Hz, 4H), 2.72 (t, *J* = 6.8 Hz, 2H), 2.68 (t, *J* = 5.0 Hz, 4H); MS (ESI, m/z): 311.1 [M+1]^+^; HRMS (EI) calcd for C_20_H_26_N_2_O, 310.2045; found, 310.2040.

#### 2.3.10 2-(4-((4-(1-phenylethyl)piperazin-1-yl)methyl)phenyl)ethan-1-ol (**12**)

White solid (ethyl acetate), yield: 78% (from compound **1**); M.p. 114°C–115°C; ^1^H NMR (400 MHz, CDCl_3_) δ in ppm: 7.58–7.09 (m, 9H), 4.23 (q, *J* = 6.6 Hz, 1H), 3.64 (br s, 8H), 3.62 (t, *J* = 6.8 Hz, 2H), 3.55 (s, 2H), 2.68 (t, *J* = 6.8 Hz, 2H), 1.12 (d, *J* = 6.6 Hz, 3H); MS (ESI, m/z): 325.2 [M+1]^+^; HRMS (EI) calcd for C_21_H_28_N_2_O, 324.2202; found, 324.2200.

#### 2.3.11 2-(4-((4-(4-methylbenzyl)piperazin-1-yl)methyl)phenyl)ethan-1-ol (**13**)

White solid (ethyl acetate), yield: 75% (from compound **1**); M.p. 112°C–113°C; ^1^H NMR (400 MHz, CDCl_3_) δ in ppm: 7.41 (d, *J* = 8.0 Hz, 2H), 7.30 (t, *J* = 8.0 Hz, 2H), 7.22 (d, *J* = 8.0 Hz, 2H), 7.02 (d, *J* = 8.0 Hz, 2H), 4.27 (s, 2H), 3.74 (t, *J* = 6.8 Hz, 2H), 3.66 (s, 2H), 3.56 (t, *J* = 5.0 Hz, 4H), 2.70 (t, *J* = 6.8 Hz, 2H), 2.64 (t, *J* = 5.0 Hz, 4H); MS (ESI, m/z): 325.1 [M+1]^+^; HRMS (EI) calcd for C_21_H_28_N_2_O, 324.2202; found, 324.2200.

#### 2.3.12 2-(4-((4-(4-methoxybenzyl)piperazin-1-yl)methyl)phenyl)ethan-1-ol (**14**)

White solid (ethyl acetate), yield: 82% (from compound **1**); M.p. 117°C–118°C; ^1^H NMR (400 MHz, CDCl_3_) δ in ppm: 7.43 (d, *J* = 8.0 Hz, 2H), 7.31 (t, *J* = 8.0 Hz, 2H), 7.23 (d, *J* = 8.0 Hz, 2H), 7.00 (d, *J* = 8.0 Hz, 2H), 4.25 (s, 2H), 3.81 (s, 3H), 3.72 (t, *J* = 6.8 Hz, 2H), 3.67 (s, 2H), 3.54 (t, *J* = 5.0 Hz, 4H), 2.72 (t, *J* = 6.8 Hz, 2H), 2.62 (t, *J* = 5.0 Hz, 4H); MS (ESI, m/z): 341.0 [M+1]^+^; HRMS (EI) calcd for C_21_H_28_N_2_O_2_, 340.2151; found, 340.2149.

#### 2.3.13 2-(4-((4-(4-fluorobenzyl)piperazin-1-yl)methyl)phenyl)ethan-1-ol (**15**)

White solid (ethyl acetate), yield: 70% (from compound **1**); M.p. 114°C–115°C; ^1^H NMR (400 MHz, CDCl_3_) δ in ppm: 7.42 (d, *J* = 8.0 Hz, 2H), 7.32 (t, *J* = 8.0 Hz, 2H), 7.21 (d, *J* = 8.0 Hz, 2H), 7.03 (d, *J* = 8.0 Hz, 2H), 4.26 (s, 2H), 3.70 (t, *J* = 6.8 Hz, 2H), 3.64 (s, 2H), 3.55 (t, *J* = 5.0 Hz, 4H), 2.70 (t, *J* = 6.8 Hz, 2H), 2.63 (t, *J* = 5.0 Hz, 4H); MS (ESI, m/z): 329.1 [M+1]^+^; HRMS (EI) calcd for C_20_H_25_FN_2_O, 328.1951; found, 328.1948.

#### 2.3.14 2-(4-((4-(4-chlorobenzyl)piperazin-1-yl)methyl)phenyl)ethan-1-ol (**16**)

White solid (ethyl acetate), yield: 72% (from compound **1**); M.p. 109°C–110°C; ^1^H NMR (400 MHz, CDCl_3_) δ in ppm: 7.43 (d, *J* = 8.0 Hz, 2H), 7.33 (t, *J* = 8.0 Hz, 2H), 7.20 (d, *J* = 8.0 Hz, 2H), 7.01 (d, *J* = 8.0 Hz, 2H), 4.24 (s, 2H), 3.72 (t, *J* = 6.8 Hz, 2H), 3.67 (s, 2H), 3.56 (t, *J* = 5.0 Hz, 4H), 2.72 (t, *J* = 6.8 Hz, 2H), 2.66 (t, *J* = 5.0 Hz, 4H); MS (ESI, m/z): 345.1 [M+1]^+^; HRMS (EI) calcd for C_20_H_25_ClN_2_O, 344.1655; found, 344.1653.

#### 2.3.15 2-(4-((4-(9H-thioxanthen-9-yl)piperazin-1-yl)methyl)phenyl)ethan-1-ol (**17**)

White solid (ethyl acetate), yield: 78% (from compound **1**), M.p. 121°C–122°C; ^1^H NMR (400 MHz, DMSO-*d*_6_) δ in ppm: 7.73–7.08 (m, 12H), 4.24 (br s, 2H), 3.67 (br s, 10H), 3.57 (t, *J* = 6.9 Hz, 2H), 2.74 (t, *J* = 6.9 Hz, 2H); MS (ESI, m/z): 417.1 [M+1]^+^; HRMS (EI) calcd for C_26_H_28_N_2_OS, 416.1922; found, 416.1920.

### 2.4 Biological evaluation

#### 2.4.1 Cell culture

LNCaP, PC-3 and WPMY-1 cells were cultured in Ham’s F-12K (PM150910) supplemented with 10% FBS (164210-50) and 1% P/S (PB180120). DU145 cells were cultured in MEM (PM150410) supplemented with 10% FBS (164210–50) and 1% P/S (PB180120). The cells were incubated at 37°C with 5% CO_2_ (Qi et al., 2022).

#### 2.4.2 Assessment of antitumor activity by CCK-8 assay

Cell proliferation was measured using the CCK-8 assay kit (Kaspers et al., 1997; Kaspers et al., 1998; Ding et al., 2010). Cells were seeded into 96-well plates (>5*10^4^) with approximately 100 ul of cell suspension per well and incubated in 37°C incubator for 4 h. Various concentrations of the compounds were then added and incubated for a further 24 h in a 37°C incubator. Finally, 10 ul CCK8 was added and incubated for 0.5–4 h, absorbance at 450 nm was determined. (Guo et al., 2021; He et al., 2022a; Hu et al., 2022).

### 2.5 AR reporter gene assay

Firefly and Renilla luciferase activities, were determined using the Dual-Glo™luciferase assay kit. RLUs were determined using the GloMax^®^96-Microplate Luminometer. IC_50_ was calculated using the GraphPad Prism 5.0 (He et al., 2022b; Qi et al., 2022).

### 2.6 Molecular docking simulation

Binding mechanism experiments performed docking analysis of the three active pockets (LBP, AF2 and BF3) (Axerio-Cilies et al., 2011; Lack et al., 2011) in AR receptors using AutoDock software (Trott and Olson, 2010). Its PDB protein data (2OZ7, 2YHD and 2YLO) was downloaded from the protein data bank (PDB) (Rose et al., 2011), and the proteins were optimized by the addition of hydrogen atoms and the removal of foreign ligands before docking. A docking space of 40 Å × 40 Å × 30 Å was constructed centered on the ligand of the AR active pocket, with compounds **11** and **12** as template molecules, docked into the optimized cavity and repeated 10 times to find a conformation of the one with the lowest binding free energy.

## 3 Results and discussions

### 3.1 Chemistry

Compounds **3–17** were synthesized by the following two-step method as depicted in Scheme 1. The compound **1** was reduced to compound **2** with BH_3_.S(CH_3_)_2_. Then, the compound **2** was heated at reflux with various piperines in an alkaline environment for 16 h.

### 3.2 Cytotoxic activity and AR antagonist activity

The *in vitro* cytotoxic activity results of the synthesized derivatives **3**–**17** against human prostate cancer lines (PC-3, LNCaP, and DU145) and the human prostate epithelial cell line (WPMY-1) were evaluated, as shown in Table 1.

**TABLE 1:** *In vitro* cytotoxicity of derivatives **3–17**.

Compd.	IC_50_ (μM)^a^			
	PC-3	LNCaP	DU145	WPMY-1
**3**	8.13 ± 0.12	6.14 ± 0.58	11.12 ± 0.76	>50
**4**	2.56 ± 0.32	4.67 ± 1.02	6.14 ± 0.24	>50
**5**	12.14 ± 0.29	3.73 ± 0.58	9.82 ± 0.24	37.51 ± 0.37
**6**	3.15 ± 0.48	6.17 ± 1.02	2.17 ± 0.72	>50
**7**	1.87 ± 0.22	5.17 ± 0.63	2.17 ± 1.12	48.26 ± 0.41
**8**	10.12 ± 0.08	4.92 ± 0.29	12.35 ± 0.16	35.53 ± 0.13
**9**	1.53 ± 0.16	4.13 ± 0.06	7.63 ± 0.12	>50
**10**	15.24 ± 0.14	7.14 ± 0.29	10.78 ± 1.14	>50
**11**	20.36 ± 0.48	11.24 ± 0.14	11.26 ± 0.27	>50
**12**	13.67 ± 1.12	7.76 ± 0.19	16.28 ± 0.63	>50
**13**	15.36 ± 0.42	22.13 ± 0.76	17.17 ± 1.02	47.14 ± 0.42
**14**	11.13 ± 0.67	16.16 ± 0.53	24.14 ± 0.47	>50
**15**	10.46 ± 1.14	18.45 ± 0.16	16.17 ± 0.62	>50
**16**	14.42 ± 0.35	16.41 ± 0.19	31.46 ± 1.21	>50
**17**	6.42 ± 0.52	5.61 ± 0.86	8.35 ± 0.28	>50
Finasteride	17.80	13.53	14.55	—
Naftopidil	42.10 ± 0.79	22.36 ± 0.61	34.58 ± 0.31	>50

^a^ IC_50_ values are taken as means ± standard deviation from three experiments.

The compounds **3–10** and **12–17** showed strong cytotoxic activity against PC-3 cells and were more potent than finasteride; the compounds **3–12** and **17** showed strong cytotoxic activity against LNCaP cells; the compounds **3–11** and **17** showed strong cytotoxic activity against DU145 cells. In addition, mono-substituted derivatives on the phenyl group (**4**, **6**, **7**, and **9**) displayed strong cytotoxic activities against all the tested cancer cells. And these compounds exhibited low cytotoxicity to normal human prostate epithelial WPMY-1 cells.

Structure-activity relationship investigation was focused on the effects of changes in different substituents on the phenyl group. For instance, compared to compound **3**, the compounds with mono-substituted group on the phenyl group (**4**, **6**, **7**, and **9**) exhibited potent anticancer activity against LNCaP, DU145, and PC-3 cells. However, Dimethyl-substituted derivative **5** displayed moderate activity against PC-3 and DU145 cells, compounds **8** and **10** also had the similar properties. Moreover, compound **6** with electron-donating group also demonstrated strong cytotoxic activities against all the tested cancer cells. In order to compare the cytotoxic activity of compounds **3–10**, the compounds **11–16** were synthesized, and the substitution of R1 and R2 groups with two phenyl groups showed high cytotoxic activity against the tested cancer cells. In summary, the introduction of this piperazine moiety contributes to its activity. Both PC-3 and DU145 cells are androgen-insensitive cell lines, but the compounds have different inhibitory activities against PC-3 and DU145 cells. The p53 is one of the most commonly mutated genes in human cancer, and the expression of p53 gene may be a key determinant of derivatives sensitivity in prostate cancer DU145 cells (Liu et al., 2013b). The literatures have reported that the p53 in DU145 cells were significantly activated by drugs, but in PC-3 cells the expression of the p53 gene was undetectable (Isaacs et al., 1991; Mashimo et al., 2000). So the compounds have different inhibitory activities against PC-3 and DU145 cells, and PC-3 cells are insensitive to derivatives.

The antagonistic activity of these derivatives against AR was assessed using luciferase assays (Xu et al., 2014; Xu et al., 2015; Zuo et al., 2017; Xu et al., 2018). As shown in Table 2, the compounds **3–10** exhibited weak antagonistic potency against AR. However, compounds **11**–**16** demonstrated relatively potent antagonistic potency (>55% inhibition).The above results were be contrary to the tested cancer cells antiproliferation activity. The results indicated that a small group introduction to the piperazine ring may be helpful for antagonistic activity against AR.

**TABLE 2:** AR antagonist activity of compounds **3**–**17**.

Compd.	AR antagonistic activity % (10 μM)
**3**	40.17 ± 0.24
**4**	32.46 ± 0.65
**5**	22.68 ± 0.72
**6**	30.75 ± 0.17
**7**	38.67 ± 0.83
**8**	25.16 ± 0.71
**9**	28.42 ± 0.25
**10**	20.47 ± 0.18
**11**	65.15 ± 0.57
**12**	68.62 ± 0.38
**13**	64.18 ± 0.45
**14**	58.88 ± 0.23
**15**	64.35 ± 0.17
**16**	60.25 ± 0.35
**17**	36.49 ± 0.79
**R1881**	N.E^a^
**Enzalutamide**	84.7 ± 1.4

^a^ N.E, no antagonistic effect.

### 3.3 Docking study

In order to better understand the binding site of derivatives targets, the docking simulation into the three binding sites of AR (LBP, AF2, and BF3) of compounds **11** and **12** were performed using AutoDock Vina software, as shown in Table 3.

**TABLE 3:** The binding affinities (kcal/mol) of compounds **11** and **12** in three binding sites of AR.

Binding site	Compound 11	Compound 12
LBP (PDB ID: 2OZ7)	−8.1	−8.5
AF2 (PDB ID: 2YHD)	−5.9	−6.0
BF3 (PDB ID: 2YLO)	−6.5	−6.6

**FIGURE 2:**
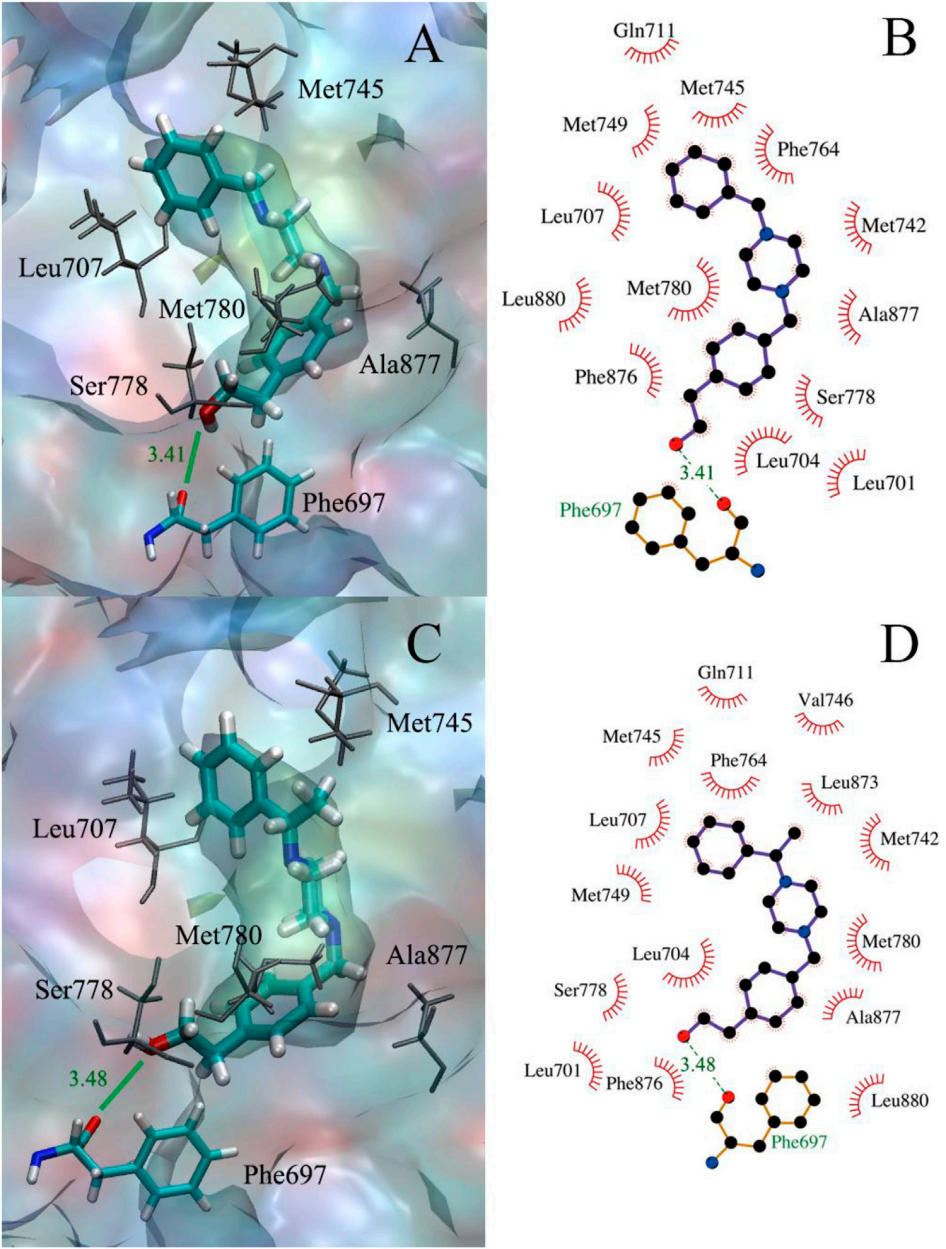
**(A,B)** The docking of compound **11** to the AR receptor; **(C,D)** The docking of compound **12** to the AR receptor.

As displayed in Table 3, the binding free energies of the compounds **11** and **12** to all three sites of the AR were calculated, both of the LBP sites had the lowest binding free energy, as measured at −8.1 and −8.5 kcal/mol, respectively. As shown in Figure 2, both compounds **11** and **12** could form hydrophobic interactions with over a dozen amino acid residues, such as Gln711, Met745, and Ala877. More importantly, they were all able to form hydrogen bonds with the amino acid residue Phe697, at a distance of between 3.5 Å.

## 4 Conclusion

In summary, in this study a series of novel hydroxyazine derivatives were synthesized and evaluated for antagonistic activity against AR and cytotoxic activity against human prostate cancer cells. The results showed that the derivative **4**, **6**, **7**, and **9** displayed strong cytotoxic activities against the LNCaP, DU145 and PC-3 cells, and compounds **11–16** showed strong AR antagonism (Inhibition% >55). The SAR results suggested that mono-substituted derivatives on the phenyl group contributed to improve the cytotoxic activity against human prostate cancer cells. These hydroxyzazine piperazine derivatives may be instructive for structural modification of novel anti-prostate cancer drugs.

## Data Availability

The original contributions presented in the study are included in the article/Supplementary Material, further inquiries can be directed to the corresponding authors.
