# Supplementary figures and images for: Synthesis, biological evaluation, and molecular docking of novel hydroxyzine derivatives as potential AR antagonists
Source: Front Chem. 2022 Nov 3;10:1053675. doi: 10.3389/fchem.2022.1053675 (PMC9671208; doi:10.3389/fchem.2022.1053675)

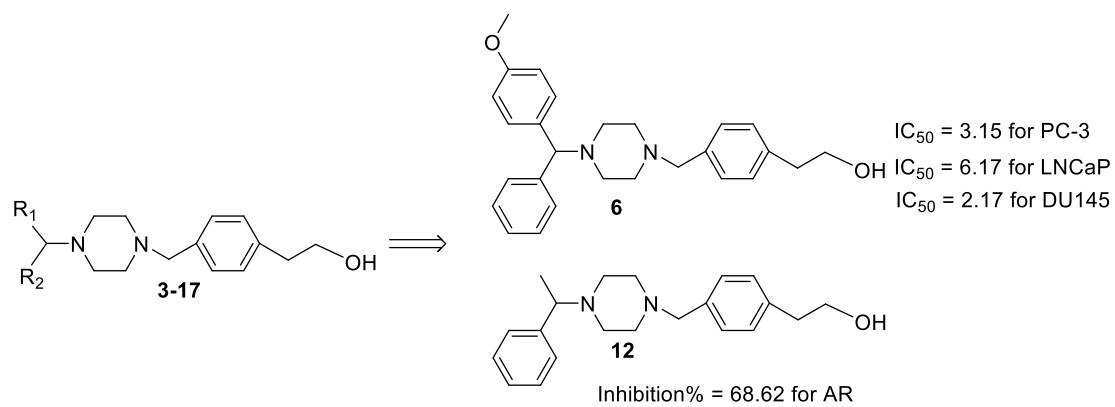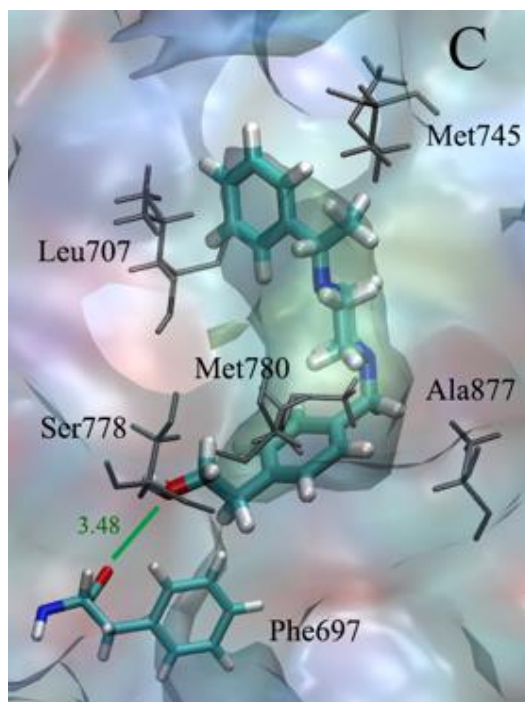

Supplement: Supplementary file 1 [file DataSheet1.PDF]
